# Trends in the Incidence of Brain Cancer: An Observational Study

**DOI:** 10.7759/cureus.72805

**Published:** 2024-10-31

**Authors:** Renee Manake, Vidith Phillips, Andrea Gangi, Jayashree Ravikumar

**Affiliations:** 1 Internal Medicine, Makerere University College of Health Sciences, School of Medicine, Kampala, UGA; 2 Internal Medicine, Division of Biomedical Informatics and Data Science, Johns Hopkins University, School of Medicine, Baltimore, USA; 3 Neurology, Universitat Internacional de Catalunya (UIC) Barcelona, Campus Sant Cugat, Facultad de Medicina y Ciencias de la Salud, Sant Cugat del Vallès, ESP; 4 Internal Medicine, Sri Varadhar Consultation Clinic, Chennai, IND

**Keywords:** brain cancer, cancer incidence, cdc-wonder, disparity, temporal trends

## Abstract

Introduction: Brain cancer is a serious global health problem, leading to increasing mortality and morbidity. Understanding the risk factors is crucial in early diagnosis and treatment. This retrospective cohort study thus aimed at assessing the temporal trends in the incidence of brain cancer based on age, gender, and race from 1999-2020.

Methods: The brain cancer incidence based on age, gender, and race were retrieved from the CDC WONDER database on May 18, 2024. Data were collected individually for each year (from 1999 to 2020), as well as for age (from <15 years to ≥ 75 years with a 10-year range in each group), gender, race, and all states, covering the overall incidence from 1999 to 2020. Additionally, data was aggregated for combined years and age, combined years and sex, and combined years and race. Statistical analysis was done via Rv4.3.2 and included the calculation of crude rates and representation of data via bar graphs and line diagrams.

Results: Our study revealed that out of a total population of 6,722,531,044 analyzed, the crude rate per 100,000 was 6.6 over 20 years. The incidence was highest in the 65-74 year age range (19.76%), among males (55.85%), and the white race (88.96%). The temporal trends overall show a rising trend in the crude rate of brain cancer, with the highest incidence in 2019, followed by a steep decline thereafter.

Conclusions: Our study found that the crude rate of brain cancer from 1999-2020 was 6.6 per 100,000, with the highest incidence in the 65-74 age group, among males, and within the white population. The overall trend showed an increase in brain cancer incidence, peaking in 2019, particularly among younger age groups, with stable or declining rates in older adults and non-white races.

## Introduction

Brain cancer is a significant cause of morbidity and mortality worldwide. It presents unique challenges due to its location in the central nervous system, impacting neurological functions and quality of life more severely than many other cancers [1].

In the United States, it is estimated that over 25,000 people will be diagnosed with brain and other central nervous system cancers in 2024, with more than 18,000 deaths [2]. Although brain cancer represents a smaller fraction of all cancer diagnoses, its impact on patients, families, and healthcare systems is profound, given its high fatality rate and the complexity of treatment.

Understanding brain cancer incidence is critical for developing effective prevention, diagnosis, treatment, and resource allocation strategies [3]. Additionally, studying brain cancer trends is essential for identifying risk factors, emerging patterns, and advancing clinical practices. Given the high mortality rates associated with brain cancer, especially in low- and middle-income countries, analyzing its incidence can offer insights into improving healthcare systems, resource allocation, and patient outcomes [4]. Furthermore, data on brain cancer incidence can help in targeting high-risk groups, refining screening programs, and developing more effective public health interventions, ultimately enhancing treatment outcomes [5].

Despite the importance of understanding brain cancer incidence, there is limited comprehensive research that spans multiple decades with detailed demographic analyses. This study aims to fill this gap by utilizing the CDC WONDER database to assess brain cancer incidence in the United States from 1999 to 2020. It provides a detailed analysis of how brain cancer incidence varies across different demographic groups and over time. By examining temporal trends, high-risk cohorts, and demographic factors such as age, gender, and race, this research offers critical insights into the dynamics of brain cancer incidence. These findings can inform future healthcare strategies, shape cancer control measures, and drive research into the causes of shifting trends in brain cancer incidence [6].

Aims and objectives

This study aims to analyze trends in brain cancer incidence and demographic disparities in the United States from 1990 to 2020 and identify any significant associations between incidence rates and age, gender, and race.

## Materials and methods

A retrospective study utilized CDC WONDER (wide-ranging online data for epidemiologic research), a user-friendly menu-driven system that facilitates access to public health data through cooperative processing and message-passing [6]. Data extraction for this study was conducted on May 18, 2024. The research involved analyzing publicly accessible de-identified data, exempting it from the requirement of ethics committee approval. This approach enables researchers to investigate epidemiological trends and patterns without accessing personally identifiable information, ensuring adherence to ethical standards in research.

On May 18, 2024, this tool was used extensively to collect detailed data from every state in the United States. The primary objective of the study was to analyze and document the incidence of brain cancer across a wide spectrum of age groups, genders, and racial demographics. Special emphasis was placed on gathering information from individual states to provide a comprehensive understanding of how brain cancer rates vary geographically within the country. The data collection spanned from 1999 through 2020, allowing for a thorough examination of trends over more than two decades.

This study focuses on brain cancer as the specific cancer site. The variables considered include temporal trends spanning from 1990 to 2020. Demographic factors encompass age, ranging from individuals aged less than 15 years to those 75 years and older, as well as both male and female genders. Additionally, the study includes individuals from diverse racial backgrounds, namely American Indian or Alaskan Native, Asian or Pacific Islander, Black or African American, and individuals identifying as White or belonging to other racial categories.

Data were collected individually for each year (from 1999 to 2020), as well as for age (from <15 years to ≥ 75 years with a 10-year range in each group), gender, race, and all states, covering the overall incidence from 1999 to 2020. Additionally, data was aggregated for combined years and age, combined years and sex, and combined years and race.

The data was exported to a Microsoft Excel (Redmond, USA) spreadsheet for further analysis. Statistical analysis was conducted using R software version 4.3.1 and ggplot2 version 3.5.0 for graph creation. Changes in brain cancer crude rates per 100,000, both overall and adjusted for age, gender, and race, as well as state-wise crude rates, were visualized using bar graphs and line diagrams.

## Results

From 1999 to 2020, the incidence of brain cancer in the United States was 443,562 cases in a total population of 6,722,531,044. The overall crude rate per 100,000 was 6.6. Table 1 shows the demographic characteristics of brain cancer patients in the United States from 1999-2020 based on age, gender, and race. The crude rate per 100,000 was highest in the age group of ≥75 years (20.1), male sex (7.5), and white race (7.4).

**Table 1:** Demographic characteristics of brain cancer patients in the United States from 1999-2020 based on age, gender, and race.

Variables	Population	Count n (%)	Crude rate per 100,000
Age			
<15 years	1331960119	40671 (9.17%)	3.1
15-24 years	932609300	19587 (4.42%)	2.1
25-34 years	917100206	28502 (6.43%)	3.1
35-44 years	927607896	37246 (8.4%)	4
45-54 years	924339992	59392 (13.39%)	6.4
55-64 years	763946196	86736 (19.55%)	11.4
65-74 years	508470217	87651 (19.76%)	17.2
≥75 years	416497118	83777 (18.89%)	20.1
Gender			
Male	3305492262	247743 (55.85%)	7.5
Female	3417038782	195819 (44.15%)	5.7
Race			
American Indian or Alaskan Native	88290475	2357 (0.53%)	2.7
Asian or Pacific Islander	373820618	11858 (2.67%)	3.2
Black or African American	914358967	31067 (7%)	3.4
White	5346060984	394583 (88.96%)	7.4
Other races	Not Applicable	3697 (0.83%)	Not Applicable

**Figure 1:**
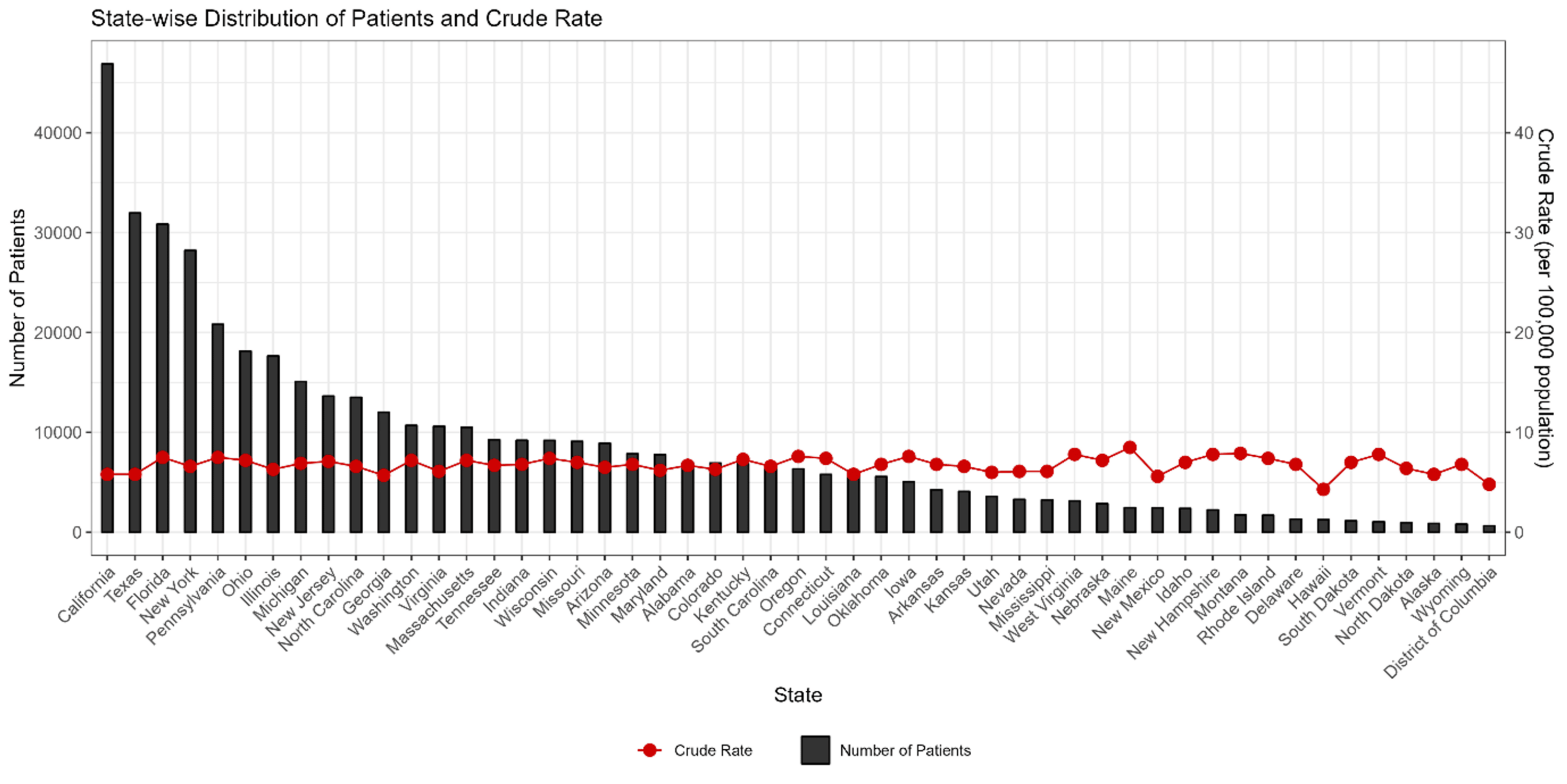
State-wise distribution of brain cancer patients in the United States from 1999-2020.

Figure 1 shows the state-wise distribution of brain cancer patients in the United States from 1999-2020. In the last 22 years, the incidence of brain cancer was highest (n=45,000+) in California, followed by Texas (n=32,000) and Florida (n=31,000). It was lowest in the District of Columbia (n=2000).

**Figure 2:**
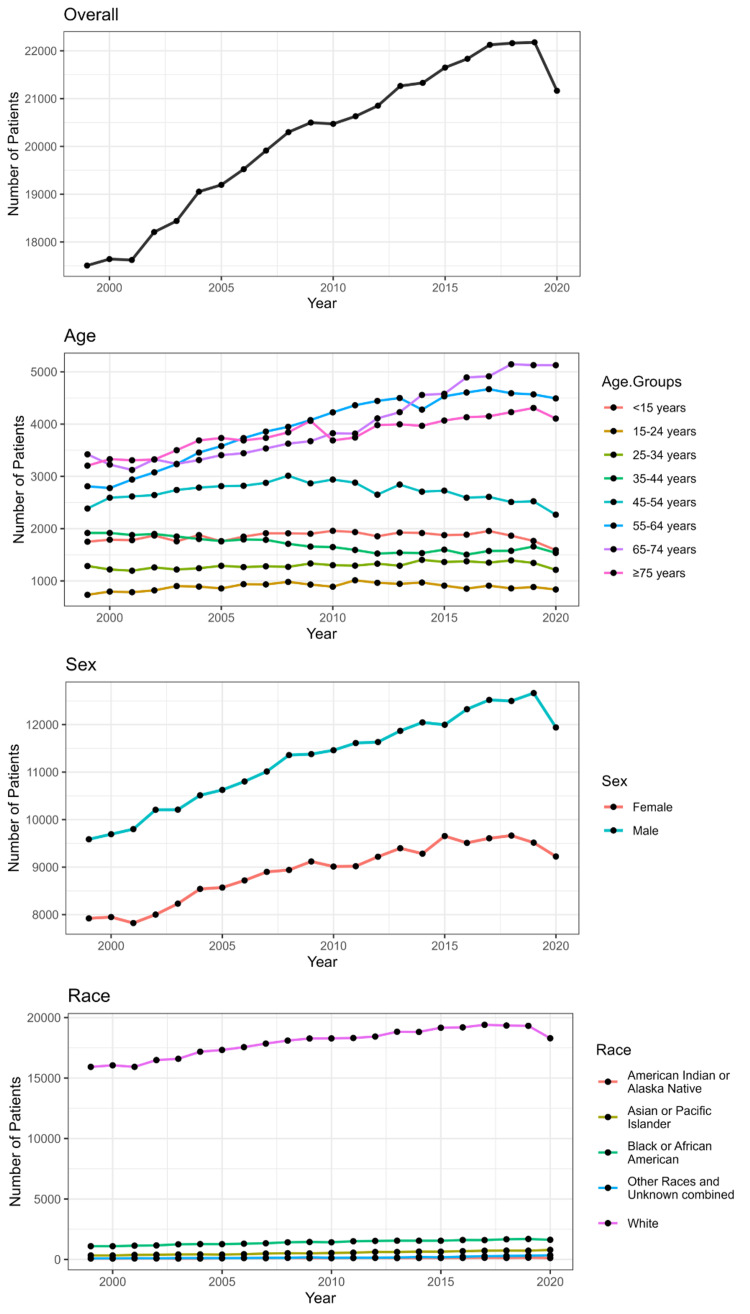
Temporal trends in the incidence of brain cancer in the United States from 1999-2020 based on age, gender, and race.

Figure 2 shows temporal trends in the incidence of brain cancer in the United States from 1999-2020 based on age, gender, and race. Overall, there is a rising trend, with the highest incidence being in the year 2019, followed by a decline in incidence. There is a rising incidence noted in the following age groups: <15, 15-24, 25-34 years. A declining/stable trend was noted in the age groups: 35-44, 44-54, 55-64, 65-74, and >75 years. A rising trend consistent with the overall trend is noted for both male and female patients. There is a slightly rising trend in the white race and a stable trend noted for all other races.

## Discussion

A retrospective original research study was conducted to examine the incidence of brain cancer in the USA over 22 years using data from the CDC WONDER database. Our study revealed that out of a total population of 6,722,531,044 analyzed, the crude rate per 100,000 was 6.6 over 20 years. The incidence was highest in the 65-74 year age range (19.76%), among males (55.85%), and the white race (88.96%). The temporal trends overall show a rising trend in the crude rate of brain cancer, with the highest incidence in 2019, followed by a steep decline thereafter.

Studying cancer incidence is crucial for understanding the dynamics of disease prevalence and guiding public health interventions. Our findings, which show varying trends across different age groups, genders, and races, align with the need to tailor health policies accordingly. Temporal trends in our study indicate a significant rise in incidence among those under 35 years, while those aged 35-74 years and over 75 years show a declining or stable trend. This rise in younger age groups and the consistently high rates in older adults emphasize the need for age-specific strategies. In contrast, a study conducted in Europe found a consistently high incidence rate in older adults but did not observe a similar increase in younger populations [7]. This suggests regional differences in factors such as environmental exposures or healthcare practices [8]. For example, higher levels of pollution and radiation exposure in certain regions can increase the risk of brain cancer, especially in older adults [9]. Lifestyle factors such as diet, smoking, and alcohol consumption vary by region and age, influencing cancer rates [10]. Differences in healthcare practices, such as screening and diagnostic methods, can also lead to higher detection rates in older populations, and genetic predispositions along with age-related genetic mutations can contribute to regional variations in brain cancer incidence [11].

Comparing gender-specific data, our study indicates a higher incidence in males (55.85%) compared to females, which is consistent with findings from similar studies conducted globally. For instance, a study in Japan reported a male predominance in brain cancer incidence, suggesting a potential genetic or environmental predisposition that warrants further investigation [12]. Furthermore, our study noted a rising trend in brain cancer incidence among both males and females, which aligns with a study from Canada that observed increasing trends in both genders, though the rates were significantly higher in males [13].

Racial disparities in brain cancer incidence are highlighted in our findings, with the white race showing the highest crude rate of 88.96%. This is consistent with a study in Australia that also found higher incidence rates in the white population compared to other racial groups [14]. The stable trend in brain cancer incidence among non-white races in our study contrasts with the rising trends seen in some parts of Asia, where rapid industrialization and lifestyle changes are thought to contribute to increasing cancer rates [15]. These differences further show the importance of considering racial and ethnic contexts in cancer research and public health planning.

Recent studies have also explored the impact of socioeconomic factors on brain cancer incidence, highlighting how disparities in income and education levels can influence disease outcomes. For example, individuals with lower socioeconomic status often have less access to healthcare resources and preventive services, which can contribute to later-stage diagnoses and poorer outcomes [16]. Additionally, research indicates that geographic disparities in access to advanced imaging and treatment options can affect incidence rates and survival [17]. Environmental factors, such as exposure to industrial pollutants and agricultural chemicals, have also been linked to increased brain cancer risk, particularly in certain regions [18]. A study examining the relationship between urbanization and brain cancer incidence found that urban areas with higher pollution levels had elevated rates of the disease [19]. Furthermore, disparities in health literacy and awareness about brain cancer symptoms can lead to delays in diagnosis and treatment [20]. Addressing these socioeconomic and environmental factors through targeted public health initiatives and policy changes may help mitigate some of the observed disparities.

The findings of our study indicate several key areas for future research and policy development. First, there is a need for policies focused on screening and early diagnosis, especially in age groups and genders with higher crude rates of brain cancer. Preventive measures should be prioritized for populations identified at higher risk, particularly older adults and males. Additionally, more research is needed to understand the underlying causes of rising trends in younger age groups and to develop targeted interventions. Policymakers should also consider racial disparities in cancer incidence and ensure that preventive and diagnostic resources are equitably distributed.

Limitations

Despite the strengths of our study, several limitations must be acknowledged. The exclusion of the latest data from 2021-2023 due to COVID-19's impact on data collection may limit the current relevance of our findings.

Additionally, details about cancer stage, grade, and outcomes were not available in the CDC WONDER database, which restricted the depth of our analysis. Future studies should aim to include more comprehensive data to provide a holistic understanding of brain cancer trends and their implications for public health.

## Conclusions

Our study found that the crude rate of brain cancer from 1999-2020 was 6.6 per 100,000, with the highest incidence in the 65-74 age group, among males, and within the white population. The overall trend showed an increase in brain cancer incidence, peaking in 2019, particularly among younger age groups, with stable or declining rates in older adults and non-white races.

Future studies should focus on understanding the role of genetic and environmental factors in brain cancer disparities across demographic groups. There is a need for policies promoting early detection and preventive strategies, especially for high-risk populations. Addressing racial disparities through equitable healthcare resource distribution and improving diagnostic and screening methods will be crucial in reducing the burden of brain cancer.
